# Deep Learning in Hematology: From Molecules to Patients

**DOI:** 10.46989/001c.124131

**Published:** 2024-10-08

**Authors:** Jiasheng Wang

**Affiliations:** 1 Division of Hematology, Department of Medicine The Ohio State University Comprehensive Cancer Center

**Keywords:** Deep Learning, Hematology, Artificial Intelligence, Whole Slide Images, Large Language Models

## Abstract

Deep learning (DL), a subfield of machine learning, has made remarkable strides across various aspects of medicine. This review examines DL’s applications in hematology, spanning from molecular insights to patient care. The review begins by providing a straightforward introduction to the basics of DL tailored for those without prior knowledge, touching on essential concepts, principal architectures, and prevalent training methods. It then discusses the applications of DL in hematology, concentrating on elucidating the models’ architecture, their applications, performance metrics, and inherent limitations. For example, at the molecular level, DL has improved the analysis of multi-omics data and protein structure prediction. For cells and tissues, DL enables the automation of cytomorphology analysis, interpretation of flow cytometry data, and diagnosis from whole slide images. At the patient level, DL’s utility extends to analyzing curated clinical data, electronic health records, and clinical notes through large language models. While DL has shown promising results in various hematology applications, challenges remain in model generalizability and explainability. Moreover, the integration of novel DL architectures into hematology has been relatively slow in comparison to that in other medical fields.

## Introduction

The public release of ChatGPT, an artificial intelligence (AI) system based on a deep learning (DL) architecture, has sparked intense discussion on the impacts of AI. This latest sensation highlights the tremendous progress made in DL over the past decade. With roots tracing back to the 1940s aiming to mimic human neuron interactions,^1^ deep learning, utilizing neural networks, has rapidly risen to prominence since the mid-2000s, due to increase in computing power and improvement in mathematical techniques.^2^ Today, DL underpins the transformative capabilities in the two major fields of AI ­– natural language processing (NLP) and computer vision (CV). Moreover, progress in the DL has been increasingly integrated into the biomedical field, enhancing various aspects of research and clinical applications.^3^

The essence of machine learning (ML) is about learning the underlying distribution of data – uncovering the intricate patterns and complex rules that govern the data.^4^ While AI and ML encompass a broader concept, DL is a subclass of ML that utilizes multi-layer neural networks to learn such distribution from vast volumes of data. In neural networks, a layer is the computational module that takes in data, performs certain mathematical operations, and then generates the transformed data. When multiple layers stack over each other, these layers work together to recapitulate the underlying distribution of data. The ‘deep’ in Deep Learning refers to having many such layers, enabling the network to learn very complex patterns.

**Figure 1.**
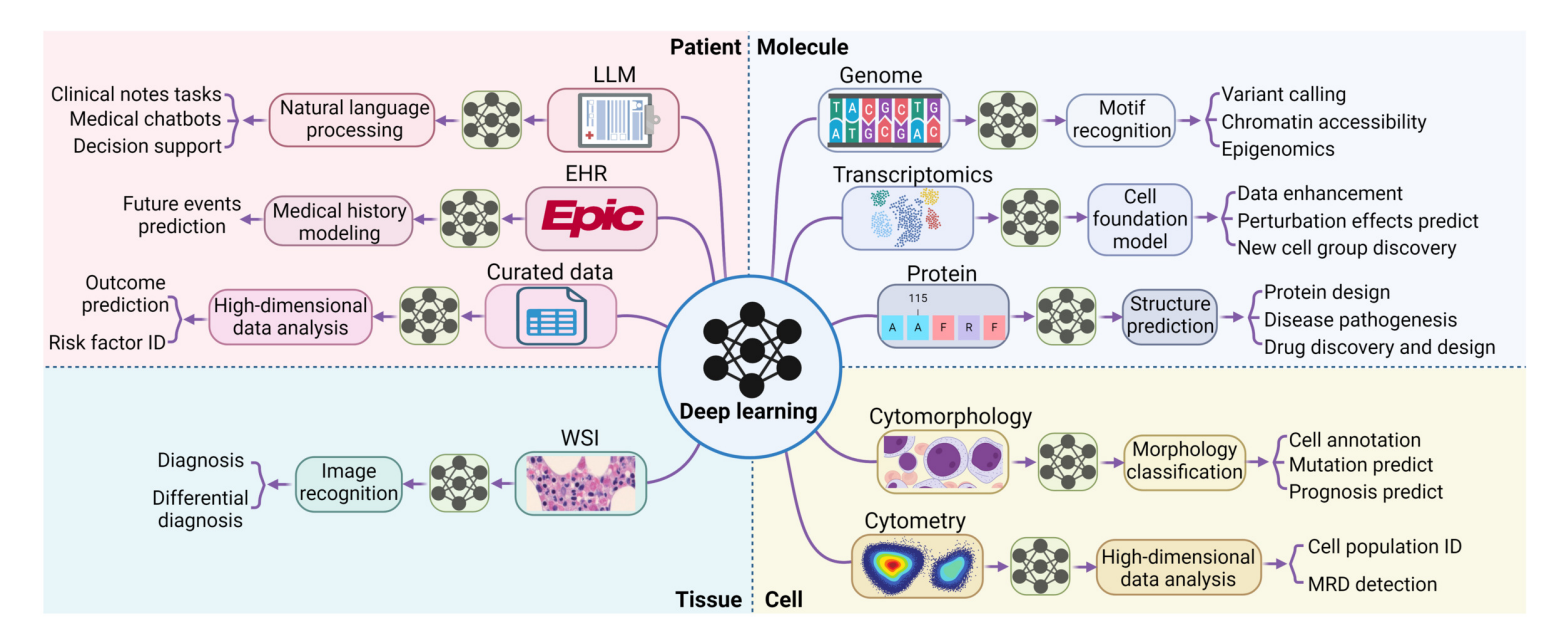
Applications of deep learning in hematology and medicine. ID, identification. MRD, measurable residual disease. WSI, whole slide imaging. EHR, electronic health records. LLM, large language model.

This review is designed to explain DL concepts and common DL models at a high level, aiming to assist hematologists in more critically appraising studies that incorporate DL techniques. It will also provide a comprehensive overview of the recent advancements in applying DL in the field of hematology, spanning from molecular to patient levels. (Figure 1) We hope to provide hematologists with a practical understanding of the field’s current capabilities and limitations.

## Deep Learning Models

### Basic process of deep learning

**Figure 2.**
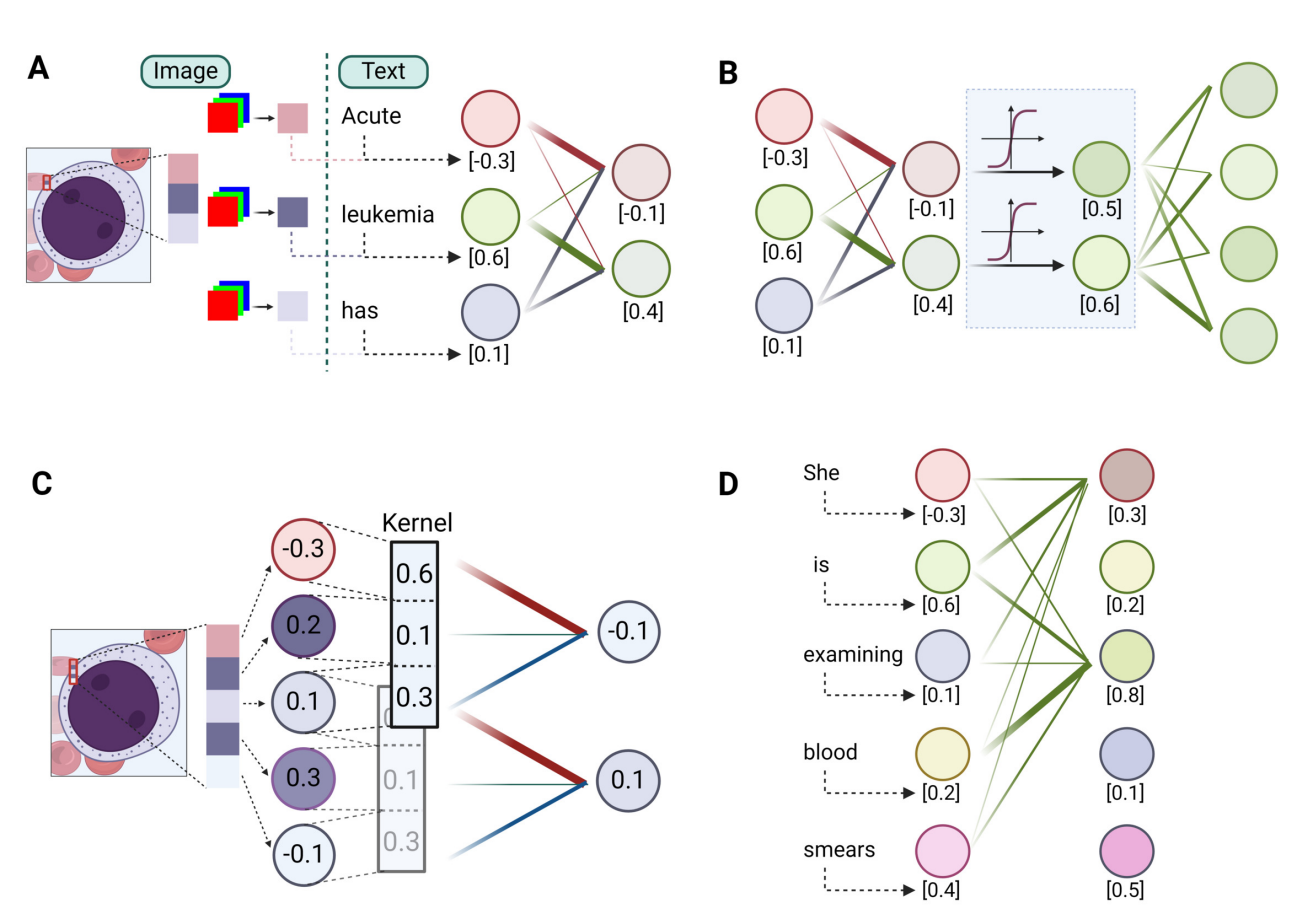
Basic modules in deep learning. **(A)** A linear layer is a key component in neural networks, where each input element (e.g., a pixel or a word) is represented by a numerical value (or values) corresponding to a node. The layer computes a weighted sum of these input nodes, multiplying each input value by a weight and summing the results. For example, given input nodes with values -0.3, 0.5, and 0.1, and weights *0.6, 0.1*, and *0.3*, the linear combination *0.6*x(-0.3)+*0.1*x(0.5)+*0.3*x(0.1) yields a new node with a value of -0.1. **(B)** An MLP (Multi-Layer Perceptron) extends the concept of linear layers by introducing a non-linear activation function (represented by the light blue shade in the figure) after each linear transformation. In this example, the sigmoid activation function maps the linear layer outputs -0.1 and 0.4 to 0.5 and 0.6, respectively. **(C)** A convolutional layer is a type of linear layer that applies a set of constant weights, called a kernel or filter, to the input data by sliding the kernel across the entire input. In the example, the kernel with weights 0.6, 0.1, and 0.3 is applied to the entire image by moving it over two pixels at a time. **(D)** A self-attention layer is a linear layer that updates each node’s value by calculating the weighted sum of all other nodes’ values, where the weights are based on the similarity between nodes. The figure shows a simplified example with two nodes, and the line thickness represents the attention weights.

In tasks like predicting the next word in a sentence or identifying an image’s content, the first step is to convert the input, be it words or images, into a digital form. This is done through data *encoding*, where words or sub-words (also called *tokens*) are represented by unique numbers, and images are broken down into pixels, also represented numerically. (Figure 2A) Each word or pixel becomes a *node*, also known as a *neuron*, the fundamental unit of neural networks. Next, the network combines these nodes using linear transformations, where each node is assigned a *weight* and summed up to create a new node. Multiple different sets of weights can be applied to the initial nodes, thus generating multiple new nodes, mimicking different ways that information can be combined. The resultant and original nodes form a *linear layer*, the basic computational unit in all DL models. (Figure 2A) By stacking multiple such layers or their variants (discussed below), a deep network is created. The network’s effectiveness is evaluated using a *loss function*, which measures the discrepancy between the network’s output value and the actual value (ground truth). Therefore, the process of training a DL network is to adjust the weights by slightly modifying them with each iteration to minimize the loss function, thereby refining the network’s predictive accuracy over time.

### Key modules in deep learning models

Built on linear layers, the multi-layer perceptron (MLP), convolutional blocks and self-attention blocks are the three most used modules in DL models. (Figure 2B-2D) At a high level, convolution blocks are mostly for image-based inputs, where they excel in extracting localized features like textures and patterns. On the other hand, self-attention blocks are tailored for sequence-based inputs, adept at identifying and emphasizing relationships and dependencies between different parts of the sequence. In contrast, MLPs serve as versatile processors, typically employed to synthesize and interpret these extracted features, effectively integrating and translating them into meaningful outputs.

The MLP, also widely known as the feedforward network (FFN) or fully connected layer, is essentially a series of linear layers interconnected with a nonlinear element known as the *activation function*. (Figure 2B) It is understood that each layer is capable of learning distinct characteristics of the input data. However, in the absence of activation functions, the network would fundamentally be a linear model, thereby constraining its capacity to handle more intricate data sets. The key purpose of the activation function is to incorporate non-linearity, enabling deep learning models to identify and learn complex patterns within the data. As a cornerstone in almost all deep learning models, the MLP plays a pivotal role in the generalization of data patterns.

Convolutional blocks are engineered for extracting features from images. The vast number of pixels in an image makes it impractical to apply a linear layer to each individual pixel. To address this, convolutional blocks use a *kernel*, which is a small-scale linear layer applied to small patches. This process, known as convolution (Figure 2C), integrates local information within these patches in a linear fashion. Similar to the way we scan an image to gather the whole information, sliding the kernel across the entire image allows the convolutional layer to extract local features from different regions. Pooling, a variant of the convolution process, outputs either the maximum or average value within a patch, rather than a linear combination. This approach enhances robustness to minor positional variations. By stacking multiple convolution blocks, the visual information of an image can be efficiently condensed into a compact form.

Self-attention blocks are designed to effectively process sequence-type data, like sentences. The key idea of self-attention is to emphasize the intrinsic relationships within a sequence. Take the sentence “She is examining blood smears” as an example. In this context, the word “examining” should have a stronger semantic connection, or more “attention”, to the word “smear” than to “blood”. This is achieved by updating the value of each word as a weighted linear combination of the values of all other words in the sentence, assigning greater weight (“attention”) to word pairs with closer relationships (Figure 2D). As a fundamental component in large language models (LLMs), self-attention is also gaining traction in computer vision tasks.

### DL models at a high level

**Figure 3.**
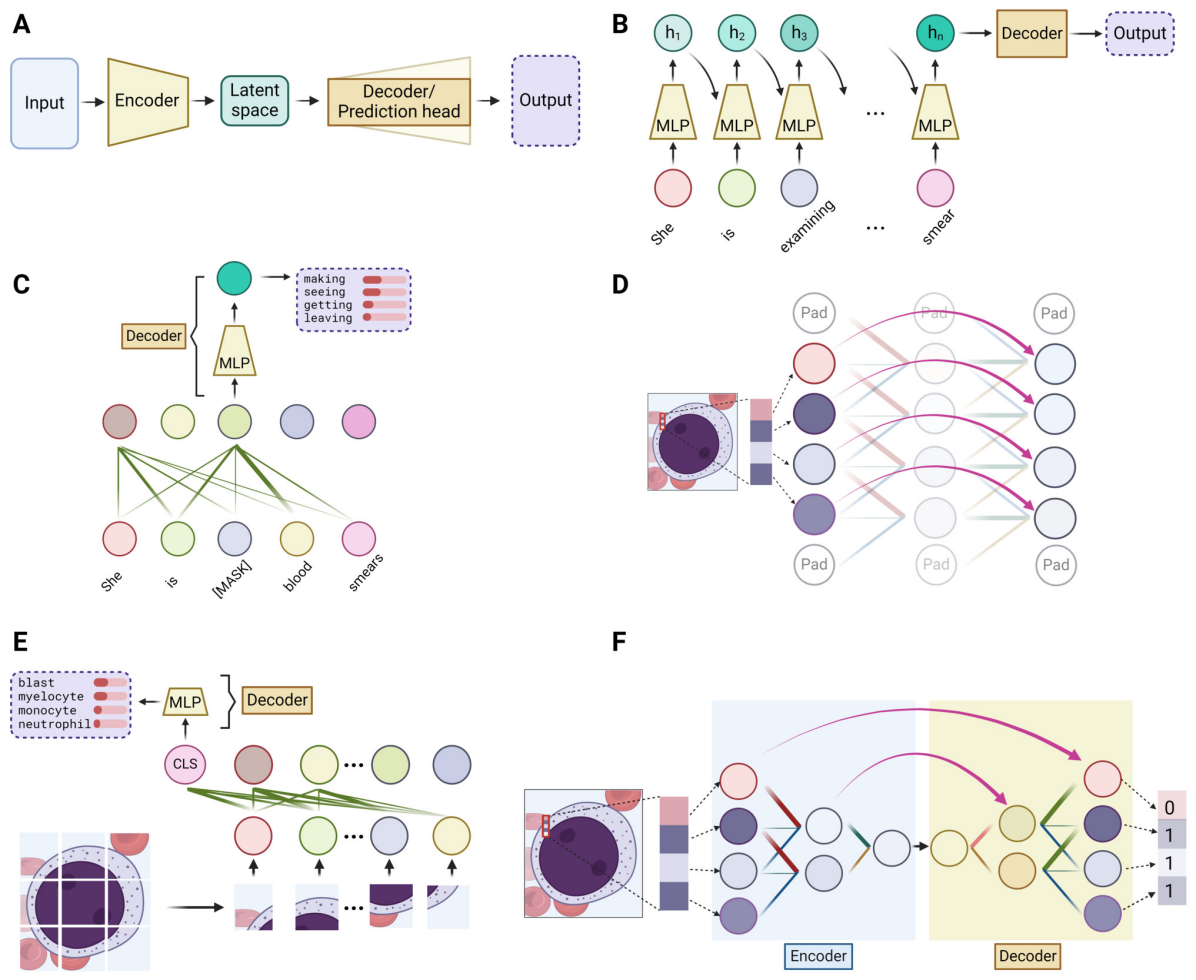
Deep learning models. **(A)** At a high level, deep learning models consist of an encoder, which transforms or condenses the input data into a more informative intermediate representation (latent space), and a decoder or prediction head, which generates the desired output from this latent representation. **(B)** Recurrent neural networks (RNNs) process data sequentially, updating a hidden state (h) at each step by incorporating information from the current input and the previous hidden state. For example, when processing the sentence “she is examining a blood smear,” the first hidden state (h_1_) is generated based on the word “she.” The second hidden state (h_2_) is then computed using the second word “is” and h_1_, allowing it to capture information from both the current and previous words. This process continues for each word in the sequence, with the final hidden state (h_n_) incorporating information from all the preceding words. **(C)** Bidirectional Encoder Representations from Transformers (BERT) utilizes the encoder component of the Transformer model, which consists of self-attention layers and multi-layer perceptrons (MLPs). During the training process, the objective is to predict randomly masked words in a sentence. Although the words are masked, the self-attention mechanism allows BERT to capture the contextual relationships between words, enabling it to infer the semantic meaning based on the surrounding context. **(D)** Residual connections in convolutional neural networks (CNN) enable the direct flow of information by skipping one or more layers, facilitating the creation of deeper networks. **(E)** The Vision Transformer (ViT) is a novel approach to image recognition tasks that adapts the Transformer architecture. In ViT, an input image is divided into small patches where self-attention is performed. A special classification token, denoted as “CLS” in the figure, is appended to the patch embeddings and participates in the self-attention process, allowing it to gather information from all patches. The output representation corresponding to the “CLS” token is then used for image classification or other downstream tasks. **(F)** The U-Net is a specialized CNN architecture that has gained popularity in medical image segmentation tasks due to its ability to perform pixel-level classification. The U-Net consists of an encoder path, which uses convolutional layers to encode it into a compact latent representation, followed by a symmetric decoder path that employs transposed convolutions to gradually restore the latent feature back to the original image resolution. Residual connections between corresponding encoder and decoder layers allow for the direct transfer of localized spatial information. In the example, the U-Net segments the blast cell from the background by assigning the value 1 to pixels within the blast and 0 to the background pixels.

At a high level, all DL models can be simplified to have an encoder and a decoder – the encoder takes the input data and condenses it into a representation which captures the essential features, while the decoder works to translate this representation into the desired output, whether it be a classification label, the next word of a sentence, or any other form of interpretable result. (Figure 3A) The encoder can be likened to the human process of learning, wherein we acquire new knowledge by distilling complex information into fundamental concepts and principles. Conversely, the decoder mirrors our application of this acquired knowledge, utilizing the simplified rules to execute specific tasks. Taking the three aforementioned modules into this context, integration of these modules can form various encoder and decoder structures. A carefully designed encoder can lead to more effective learning of the data, which is often a primary focus in deep learning models. The complexity of the task dictates the structure of the decoder – for simpler tasks such as classification or next-word prediction, an MLP would simply suffice. However, for more complex tasks like image segmentation or language translation, a combination of different modules is typically employed.

In the following sections, we will introduce key DL models in the two major fields of artificial intelligence – natural language processing (NLP) and computer vision (CV). This is particularly relevant since DL applications in hematology predominantly stem from advancements in these two fields.

### DL models in natural language processing

At its core, NLP involves processing sequence-type data, as a sequence of words forms a sentence. Traditionally, the *Recurrent Neural Network* (*RNN*) was the go-to method for encoding sequences, until the advent of the Transformer model. The fundamental concept of a simple, or “vanilla”, RNN is to devise a method for passing information through a sequence as each component is processed sequentially. This is achieved by employing a set of evolving values, known as the *hidden state*. The hidden state retains the information from all previously processed components and updates itself with each new component of the sequence. This integration of the previous hidden state and the current sequence component, facilitated through an MLP-like structure, generates the new hidden state. Therefore, as an encoder, RNN effectively encodes the entire sequence into this final hidden state.(Figure 3B) An improved variant of the vanilla RNN, known as *Long Short-Term Memory network* (*LSTM*), has gained popularity for its enhanced ability to handle longer sequences.^5^

Since 2017, the field of NLP has undergone a significant transformation with the introduction of the Transformer model.^6^ Traditional RNN models encode sequence-type data slowly, as they integrate information one component at a time through updating the hidden state. The Transformer model addresses this limitation by applying the self-attention module to each sequence component simultaneously, allowing for parallel rather than sequential integration of information. Selectively utilizing core elements of the Transformer architecture, which principally consists of stacks of self-attention modules linked to an MLP, the Generative Pre-trained Transformer (GPT) models and Bidirectional Encoder Representations from Transformers (BERT) models stand out as two of the most prominent large language models (LLMs).^7,8^ (Figure 3C) Another key factor contributing to the success of LLMs has been the advancement of graphic processing units (GPUs), enabling large-scale parallel training.^9^ Empirical evidence suggests that the effectiveness of LLMs depends not only on the model size (number of trainable weights) but also on the volume of data used for training.^10^ Modern LLMs typically boast tens to hundreds of billions of parameters and are trained on vast corpora, encompassing hundreds of billions of words.

### DL models in computer vision

In CV, the encoder part of DL models is typically referred to as the *backbone* network, which is dedicated to extracting image features. This backbone is then integrated with MLPs to perform simple downstream tasks, such as classification. More complex tasks, such as objective detection with bounding boxes and image segmentation, require extensive postprocessing steps or a dedicated decoder structure.^11,12^

Traditionally, the backbone of DL models in CV has been convolution module-based deep neural networks, commonly known as *Convolutional Neural Networks* (*CNNs or ConvNets*). Variations in the arrangement and the total number of stacked convolution modules differentiate well-known vanilla CNNs, such as *AlexNet* and *VGG*.^13,14^ An important advancement in CNNs is the development of the *Residual Network* (*ResNet*), which employs a unique mechanism called *residual connections* that allow the output of one layer to skip some layers and be added directly to the output of a later layer.^15^ (Figure 3D) This approach enables the training of much deeper models by ensuring efficient flow of information through the network.

Since the introduction of the Transformer model in NLP, self-attention modules have attracted significant interest as a potential backbone structure. However, the high pixel count in images poses a computational challenge for calculating self-attention across all pixels. To address this, the *Vision Transformer* (*ViT*) model segments an image into hundreds of patches. This approach allows the application of self-attention among individual patches rather than to each pixel, thereby reducing the computational load.^16^ (Figure 3E) However, ViT requires extensive data for training to outperform CNN-based models. Another attention-based model is the Shifted Window Transformer (*Swin Transformer*).^17^ Drawing inspiration from Convolutional Neural Networks, the Swin Transformer initially divides the original image into small patches. It then applies self-attention within each patch, akin to how a kernel operates on patches in CNNs. Subsequently, to amalgamate information from different patches, the Swin Transformer uses shifted windows and progressively combines smaller patches into larger ones. This approach facilitates a multi-scale representation, mirroring the hierarchical structure typical of CNNs.

Image segmentation, delineating object of interest within an image down to the pixel level, is particularly useful in hematology because it isolates blood cells from the noisy background in smear or biopsy samples for the subsequent identification of the cell morphology. This task requires pixel-level prediction, and a decoder structure is typically required. A widely used network for image segmentation is the *U-Net*, which utilizes a CNN encoder to decrease the spatial size, followed by a decoder that progressively restores the CNN output to the original size by reversing the operation of convolution (known as transposed convolution).^12^ (Figure 3F) This upscaling process results in the mapping of features learned by the CNN back onto the image’s original pixel grid, producing per-pixel predictions that determine whether each pixel is part of the object of interest. The recently introduced Segment Anything Model (*SAM*) features the ViT as its encoder and employs a combination of attention modules and transposed convolution in its decoder.^18^ Trained on an extensive dataset, this model has outperformed networks based on the U-Net architecture.

### Training DL models

The objective of training a DL model is to minimize the difference between the network’s output and the actual value (the ground truth) for a given input, or the loss function, by adjusting the model’s weights. This ground truth can be manually annotated, such as determining a classification label for an image or delineating an object’s segmentation border, a process characteristic of *supervised learning*. However, due to the extensive human effort involved, these datasets tend to be small. *Unsupervised learning*, also known as *self-supervised learning*, rather than predicting manually assigned labels, focuses on predicting individual data point itself, which effectively serves as its own “ground truth”; this guides the model to uncover the underlying structure of the dataset. A prominent unsupervised learning architecture is the autoencoder. It typically uses MLPs to compress the original data into a smaller, condensed form, known as the latent space. The autoencoder then uses another set of MLPs to expand the compressed data back into its original form. The compression process encourages the model to learn the most salient features of the data, much like how we learn new information by summarizing key points.

In essence, various training methodologies in unsupervised learning revolve around how to effectively formulate a *pretext task*. In autoencoders, the pretext task is to minimize the difference between the input and its reconstruction. In NLP models such as *BERT*, the pretext task involves masking certain words in a sentence and training the model to predict these hidden words based on the surrounding context.^8^ This method allows language models to grasp the underlying rules of a language, akin to how cloze tests facilitate language learning in humans. Unsupervised learning in CV is predominantly achieved via contrastive learning, which is under the idea that differently augmented views of the same original image should be labeled as the same, or “positive samples” serving as the ground truth, while views of different images are all different and should be labeled as “negative samples”. The two classic training methods in CV contrastive learning are called *MoCo* and *SimCLR*, where the pretext task is to distinguish between pairs of similar (positive) and dissimilar (negative) images.^19,20^ An improvement upon the above method involves only using the positive samples, as the pretext task is re-designed to minimize the difference of two differently augmented views of the same image. Example techniques include *BYOL* and *DINO*.^21,22^ Lastly, inspired by the success of NLP models like BERT, which use masked word prediction as a pretext task, a similar concept has been adapted in CV. For instance, the masked autoencoder (*MAE*) technique involves randomly masking a significant portion of an input image and training a model to predict and reconstruct the missing patches.^23^

However, the pretext tasks in unsupervised learning models, like reconstructing missing patches in an image, typically differ from the desired end tasks, such as identifying objects within an image. Nonetheless, the encoder part of these models has inadvertently learned efficient methods for feature extraction. Consequently, we can repurpose these encoders by pairing them with various decoders tailored to specific downstream tasks. In such scenarios, it is often sufficient to train only the decoder. This approach of leveraging a pre-trained model for new applications is commonly referred to as *transfer learning*. When a pre-trained model is large-scale and demonstrates strong performance across a variety of transfer learning tasks, it is often referred to as a *foundation model*, such as GPT. A closely related concept of transfer learning is *fine tuning*, where instead of training just the decoder, the entire model, including the pre-trained encoder, undergoes additional training to better adapt to the specific requirements of the new task.

For CV models, another popular training method is known as *weakly supervised learning*, which has been adopted mostly in analyzing whole slide images (WSIs) in pathology. Given the large size of a WSI, annotation of every single cell or every patch within a slide is extremely labor intensive, but the label for the whole slide is often known. To learn the label of individual patches, a commonly used method in weakly supervised learning is *multiple instance learning* (*MIL*). This approach aggregates the information from each small patches in a WSI to predict the overall label of the slide.^24,25^ Even with only the WSI label available, this training approach is still effective in learning the labels of individual patches. This is because, for instance, when training a model to distinguish between slides with blasts and those without, the model learns the characteristics of “no blasts” from all the patches in a WSI labeled as “no blasts”, since none of the patches should contain blasts.

## Deep Learning at the Molecular Level

### DL in genome

**Figure 4.**
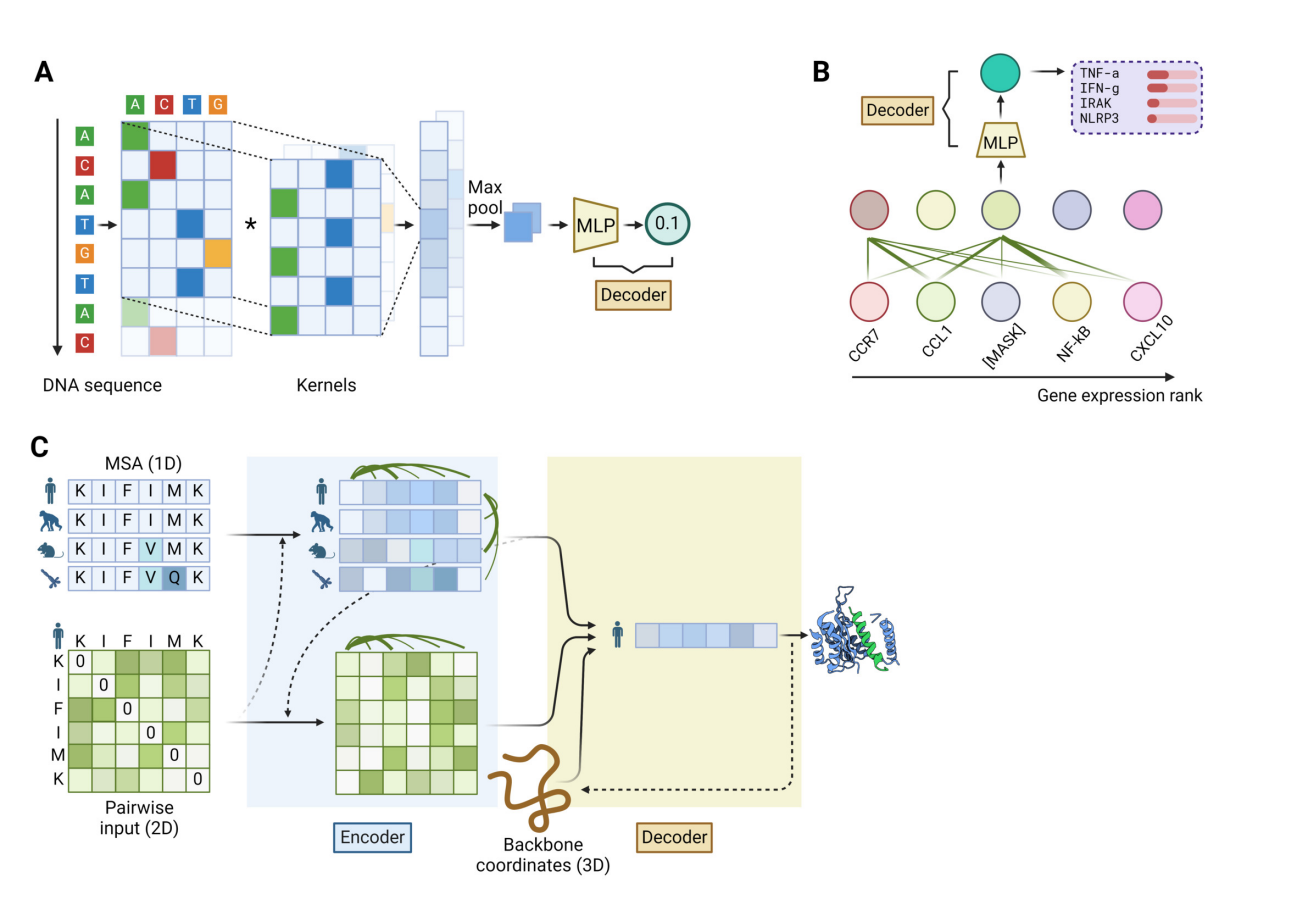
Deep learning models on molecules. **(A)** A common architecture of using convolutional neural networks (CNNs) to investigate genome. First, the DNA sequence is converted into a two-dimensional matrix with 4 columns, where each row corresponds to a nucleotide position and each column represents one of the four nucleotides (ACTG). Next, kernels with a fixed width of 4 scan through the sequence, capturing specific local patterns (motifs) in the DNA sequence. This step is followed by max pooling, which selects the nodes with the most prominent features from each convolutional output. Finally, a multi-layer perceptron (MLP) decoder calculates the probability of the presence of the target motifs. **(B)** A self-attention mechanism for single cell transcriptome analysis. The expression levels of genes are ranked from high to low, forming a “sentence” with each gene representing “words”. A BERT model is trained to predict the name of the masked genes. **(C)** A simplified demonstration of alphafold-2 (AF2) for protein structure prediction. It uses multiple sequence alignments (MSA) of the target protein across different species (shown in light blue) to capture evolutionary information, as spatially close amino acids tend to co-mutate. MSA is a 1D input containing only sequence information. AF2 applies self-attention to the MSA to integrate information across sequences and species. Additionally, AF2 uses a 2D pairwise input (shown in light green) representing the predicted pairwise spatial distances between amino acids, which is initially derived from prior knowledge. The pairwise input is further refined in the encoder by integrating MSA information, geometric constraints, and self-attention mechanism. The decoder of AF2 takes the processed MSA, pairwise distances, and initial 3D backbone coordinates as inputs. It iteratively refines the backbone coordinates and generates an updated representation of the sequence, which can be used to predict the positions of side chains and complete the protein structure prediction.

The inherent complexity and large volume of genomics data render them particularly suitable to DL models. The primary tasks in genomics include identifying non-coding regulatory elements, such as promoters, enhancers, and transcription factor binding sites, as well as interpreting the effects of non-coding single-nucleotide polymorphisms (SNPs). In 2015, two seminal studies and their proposed models, named DeepBind and DeepSEA, aimed to tackle the above problems, respectively.^26,27^ These models laid the foundation for many current DL-based approaches by employing a shared methodology. First, the one-dimensional DNA sequence was converted into a two-dimensional representation, akin to a “picture”, where the added dimension comprised four “pixels”; each pixel symbolized one of the four nucleotide bases (ACTG) at a specific position. Subsequently, a convolutional neural network was deployed to extract sequence features, which were then linked to an MLP to formulate predictions (Figure 4A) Both models outperformed non-DL based tools at the time. Recently, the application of Transformer-based methods to DNA sequences, inspired by their success in processing human language data, has been explored. The DNA-BERT model, for instance, interprets groups of 3 to 6 adjacent nucleotides as a single “word”. In this approach, the primary task of unsupervised learning is to predict these “words” when they are masked in a DNA sequence. After undergoing fine-tuning for specific downstream tasks, DNA-BERT demonstrated improved performance over CNN-based models such as DeepBind and DeepSEA, across a range of metrics.^28^ However, the direct application of DL models to predict variant effects in hematology is limited for several reasons. First, the experimental validation of causal variants continues to be the gold standard and can be readily conducted when the SNP data are not extensive.^29^ Second, recently developed machine learning methods, such as regBase, which integrates predictive outcomes from a variety of non-DL and DL-based models, have yielded superior results compared to employing a single DL-based model alone.^30^ Consequently, these integrated approaches are more frequently utilized.^31^

### DL in karyotyping

Karyotyping through chromosome banding analysis remains the definitive method for detecting cytogenetic abnormalities, despite being both time-consuming and labor-intensive. The advent of DL in automating karyotyping reflects its broader progress within the field of CV. Initially, before CNNs were introduced, the process required extensive manual annotations and significant domain knowledge to extract chromosome features manually, which were then classified using an MLP.^32^ However, the emergence of AlexNet marked a turning point, enabling CNN-based models to achieve over 90% accuracy in classifying normal chromosomes.^33^ More recent advancements, particularly through the implementation of residual connections and deeper CNN architectures, have further improved accuracy, pushing it beyond 95% in classifying normal chromosomes.^34^ The workflow typically starts with software-assisted automatic preprocessing of metaphase images, involving segmentation and organization into karyograms. The processed images are subsequently analyzed by CNN for the classification of chromosomes. Despite these advancements, detecting chromosome aberrations is still challenging due to their complexity and the rarity of some aberrations during the training process. Recently, models based on self-attention mechanisms, such as the ViT, have been employed to address this challenge. By initially pre-training on a large dataset focused on classifying normal chromosomes, and subsequently fine-tuning on a smaller dataset containing aberrant chromosomes, ViT-based models have achieved accuracies exceeding 95% in identifying chromosomal aberrations.^35^

### DL in transcriptomics

Gene expression profiling (GEP) data, derived from bulk RNA-sequencing or microarray techniques, are inherently “high dimensional.” This is because each gene’s expression level introduces a unique “dimension” to the analysis, making the dataset well-suited for machine learning techniques. In this scenario where the data structure is relatively straightforward, traditional machine learning methods, such as Lasso regression (a specialized form of linear regression), tree-based algorithms (including random forest and gradient boosting trees), and Support Vector Machines (SVM), often perform comparably or even better than DL models like MLP.^36^ For instance, a study aimed at distinguishing between acute myeloid leukemia and other forms of leukemia using peripheral blood GEP data demonstrated that both classical machine learning techniques and neural networks could achieve accuracy rates exceeding 95%.^37^

However, data obtained from single-cell RNA sequencing (scRNA-seq) encompasses RNA expression information from thousands of individual cells, presenting both a massive scale and complexity that make it ideal for DL-based methods. One application of DL in scRNA-seq is in data processing. scRNA-seq data are inherently noisy, not only because current techniques capture less than 30% of all transcripts leading to dropout events for specific genes, but also because the data exhibit variability from batch to batch. This variability introduces the well-known batch effect, further complicating the analysis.^38^ Various DL models have been developed to tackle these problems.^39^ Among these, scVI stands out as a widely adopted tool that employs a variational autoencoder (VAE) to learn a low-dimensional latent representation of the data, effectively capturing its key patterns.^40^ The normalized distribution characteristic of the latent space in a VAE enables it to manage missing values and dropout events, while simultaneously mitigating batch effects, because it smooths out variations that arise from different batches.^40^

In addition to data processing, DL methods are also highly effective at modeling cell behavior based on gene expression. For example, one study aimed to identify the counterpart of hematopoietic stem cells (HSCs) within induced pluripotent stem cells (iPSCs).^41^ In that research, an MLP was trained to identify HSCs from human fetal liver cells based on the differential expression of thousands of genes. Once trained, this MLP model could then be applied to pinpoint HSCs population within iPSCs, utilizing the expression of the same gene set.

Building upon the success of foundational models in NLP, recent initiatives have sought to develop large transformer-based models tailored to scRNA-seq data, treating genes and cells in a manner analogous to words and sentences.^42–44^ Inspired by unsupervised training techniques used in NLP transformers, these “single-cell foundation models” are trained on expression data from billions of individual cells. During the pre-training stage, the models learn to predict masked genes and their relative expression levels. (Figure 4B) Much like how LLMs learn word relationships and grammar, these cell models develop an understanding of gene interactions and biological patterns. For instance, the geneformer model, when fine-tuned with a specialized dataset of diseased cardiomyocytes, successfully identified genes whose alterations could lead to cardiomyopathy.^43^ While this concept is intriguing and the preliminary results are promising, the efficacy of these models compared to existing scRNA-seq analysis methods warrants further evaluation.^45^ As of now, their use in hematology has not been documented. However, they hold potential for various applications, such as discovering unique cell groups, identifying gene expression patterns specific to diseases, predicting how cells might respond to treatments, and revealing new cell states associated with disease development.

### DL in protein structure predictions

DL has revolutionized the field of protein structure predictions, with the success of AlphaFold2 (AF2)^46^ and other models inspired by AF2, including RoseTTAFold and ESMFold.^47,48^ Leveraging the achievements of preceding models, AF2 integrates modules and design tricks proven to enhance protein prediction, resulting in a complex architecture.(Figure 4C) AF2 begins by constructing a multiple sequence alignment (MSA), which is a widely used method in protein prediction tasks. An MSA aligns homologous protein sequences across different species. This is helpful in protein structure prediction because amino acid residues in close spatial proximity tend to co-evolve in different species. Simultaneously, a pairwise input is initiated, which is a 2-dimensional (2D) table representing the spatial distances between each pair of amino acid residues within a protein. Next, the encoder of AF2 employs self-attention modules to process relational information between amino acids. This integration occurs in two domains: the sequence space (1D) derived from the MSA, and the structural space (2D) derived from the pairwise input. These self-attention modules allow AF2 to understand the relationships between amino acids in both their sequence and spatial arrangements. Additionally, the encoder incorporates geometric rules to ensure the encoding of physically plausible protein structures. The AF2 decoder integrates encoded sequence, pairwise data, and initial protein backbone coordinates (3D information) to determine the 3D coordinates of the backbone and side chains. This process involves synthesizing 1D, 2D, and 3D information with geometric rules for precise protein structure modeling.

Although AF2 can achieve sub-atomic resolution accuracy, it faces several challenges. These include its inability to predict structures of multimeric proteins, proteins with post-translational modifications, or those associated with ions or cofactors; additionally, AF2 performs less effectively in predicting proteins that have mutations or disordered regions.^49,50^ Moreover, it has poor performance in modeling ligand and drug binding sites. This is likely because AF2, being primarily designed for protein structure prediction, may not capture the subtle but critical features of the protein’s active site where ligands bind.^51^ These limitations have restricted the application of AF2 in fields like drug discovery and studying the impact of protein mutations. Notably, recent studies have proposed improved models based on AF2 to address these problems. These include models that can predict the structure of protein-nucleic acid complexes, identify the effects of pathogenic mutations, or model multimeric protein structures.^52–54^ However, these models require further validation across diverse datasets. For instance, Chabane *et al.* evaluated *AlphaMissense*,^53^ essentially a version of AF2 fine-tuned to detect pathogenic variants, on sequencing data from 686 samples of patients with hematological malignancies.^55^ Out of 853 variants known to be pathogenic from the literature, *AlphaMissense* correctly identified 80% of them.^55^ Therefore, given their current performance, while these tools are promising for generating hypotheses, experimental verification remains essential for confirmation.

DL-generated protein structure prediction has been utilized in hematology to elucidate biological functions. For instance, Frunt *et al.* used AF2 to show how Factor XII, which lacks a known crystal structure, binds to anionic surfaces and exposes its activation site.^56^ In a separate study, Renella *et al.* discovered a novel germline mutation in *SEPT6*, associated with severe neutropenia and dysmyelopoiesis in an infant.^57^ To investigate the mutation’s pathogenic role, they used AF2 to illustrate how this mutation alters the structure and impacts the dimerization of the SEPT6 protein.^57^

## Deep Learning at the Cell Level

### DL in cytomorphology

Automated analysis of peripheral blood smear (PBS) or bone marrow smear (BMS) is an early application of DL in hematology. Several deep learning-based digital cell morphology systems, like CellaVision DM96 and Scopio Labs X100, have gained US FDA Class II medical device approval for PBS analysis.^58^ These systems typically achieve over 90% accuracy in classifying normal white blood cells (WBCs).^59,60^ In the clinical workflow of identifying WBCs, these systems start by scanning a blood smear, specifically targeting the monolayer region where cells are spaced closely but not overlapping. The systems then segment the WBCs in the region into patches based on manually engineered features. These patches containing individual WBCs are displayed on a screen, and the system “pre-classifies” the cells as normal or abnormal. Finally, trained technicians verify the pre-classifications. Both CellaVision and Scopio utilize color and shape-based segmentation to isolate WBCs. CellaVision then applies image processing techniques to extract key features from each cell, such as size, shape, and color, before employing a MLP for the final step of classification. On the other hand, Scopio opts for CNN-based methods to classify the cells.^61^ The specific details of their model architectures remain undisclosed due to proprietary considerations. Nonetheless, a study showed that various pre-trained CNN models can all achieve approximately 90% accuracy in WBC identification.^62^ However, CellaVision struggles with accurately identifying rare abnormal cells in peripheral blood, such as plasma cells and lymphoblasts, due to insufficient training data points.^63^

**Figure 5.**
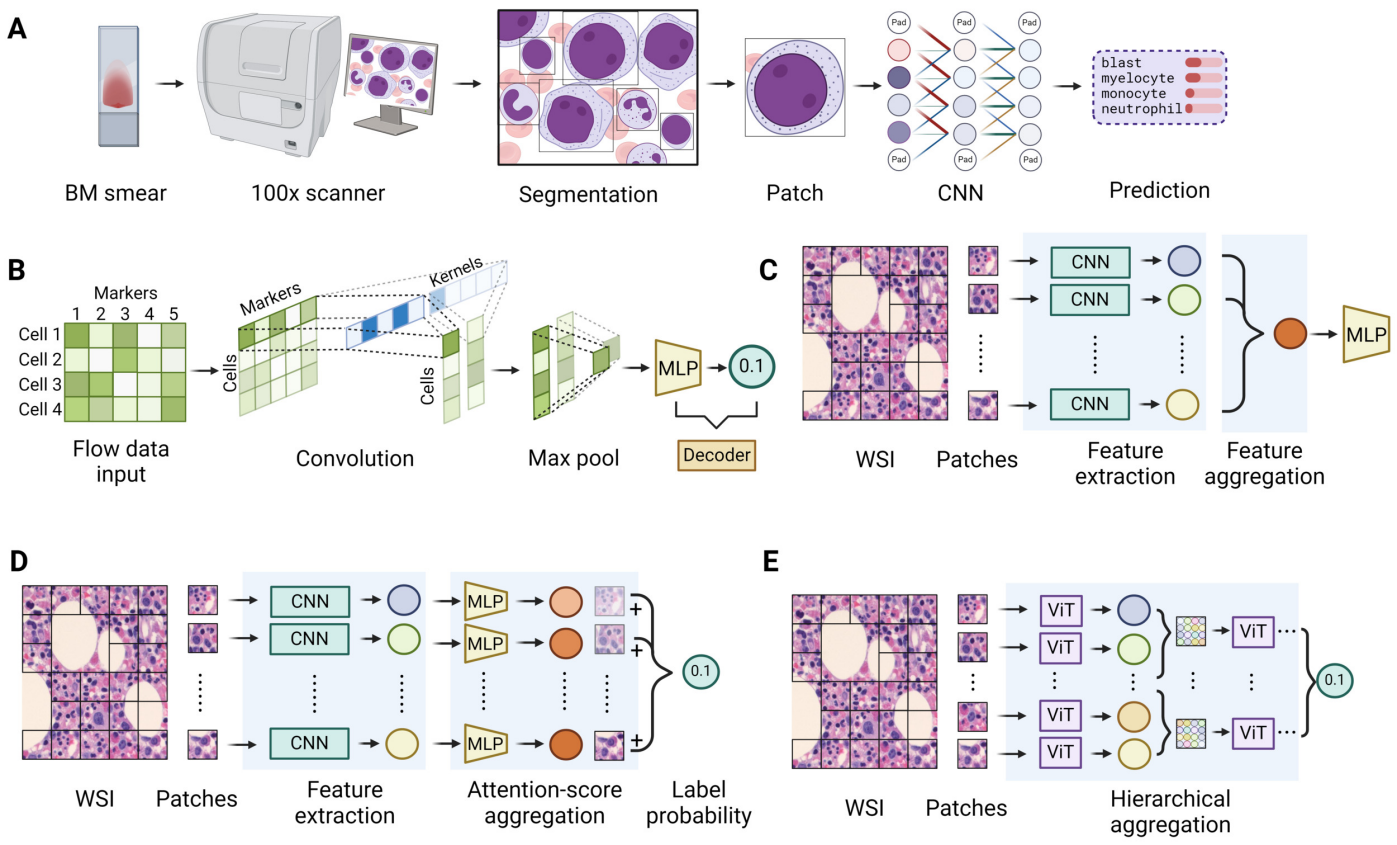
Deep learning models on cytomorphology and whole slide image (WSI). **(A)** A workflow of automatic white blood cell (WBC) annotation on bone marrow smear samples. First, the smears are scanned and magnified, and regions with appropriate cell density are identified. Next, object detection algorithms, either based on hand-crafted features or pre-trained deep learning networks like Faster R-CNN or YOLO, are applied to draw bounding boxes around individual WBCs. Finally, a convolutional neural network (CNN) is used to classify the specific cell type within each bounding box. **(B)** A deep learning model for automatic analysis of flow cytometry data takes the raw data table, where rows represent individual cells and columns represent marker fluorescence intensities, as input to a CNN. The CNN kernels have the same width as the number of markers and a height of one, summarizing marker information for each cell. A max pooling layer then selects the most prominent cells. Finally, an MLP prediction head outputs the probability of the presence of cells with specific marker combinations. **(C)** The general framework for WSI analysis. First, the high resolution WSIs are divided into smaller patches, after which feature extractors, such as CNNs or ViTs, are applied to each patch to obtain meaningful representations. Next, a feature aggregation step, using techniques like pooling or attention score-based methods, combines the patch-level features into a unified representation. Finally, the aggregated features are passed through a prediction head to generate the desired output, such as class probabilities. **(D)** WSI analysis using an attention score-based aggregator. After extracting features from individual patches using a CNN, MLPs are used to generate attention scores indicating the significance of each patch for classification. These scores enable the creation of a heat map on the WSI, highlighting the most informative regions. The patch-level features are then weighted by their attention scores and summed to generate the overall probability of a certain label. **(E)** WSI analysis using a hierarchical aggregator. At the lowest level of hierarchy, a ViT is used to extract features from the pixels of individual patches. The extracted patch-level features are then treated as “pixels” for the next level of the hierarchy, forming higher-level “patches”, where a ViT can be applied again to extract the features. This process can be repeated and at the highest level, a final ViT extracts the slide-level representation.

Automated BMS analysis is more complex than PBS analysis due to several factors. BMS contains a wider range of cell types, including both normal and abnormal cells, and faces challenges in cell segmentation because of the variable cell sizes, cell adhesion, and artifacts like dye impurities. Consequently, larger annotated datasets are necessary for effective training. Moreover, an additional module is typically needed to segment cells of interest. In one of the most extensively tested systems, Morphogo, nucleated cells are segmented using a traditional machine learning method, a decision tree based on the distribution of color range. These segmented cells are then input into a 27-layer CNN connected to an MLP for label generation.^64^ (Figure 5A) The system can reach over 95% accuracy in identifying normal mature and immature granulocytes and erythrocytes, as well as blasts.^65,66^ Other BMS analysis models employ pre-existing DL-based segmentation tools which have already been widely used in computer vision tasks. Tools such as YOLO (You Only Look Once) and Faster R-CNN (Region-based Convolutional Neural Networks) are utilized to precisely detect and segment target cells.^67–72^ A particularly challenging scenario involves analyzing cell morphology in bone marrow biopsy samples, where cells are densely clustered. In a study by Sirinukunwattana *et al.,* which focused on differentiating various myeloproliferative neoplasms (MPNs) through megakaryocyte morphology in bone marrow trephines, the U-Net was employed for pixel-level segmentation of megakaryocytes from the surrounding tissue.^73^ This approach yielded an impressive AUC of 0.98, distinguishing between reactive and MPN samples. This method has also been adopted by other studies to classify bone marrow cells based on morphology.^74,75^ While the models mentioned previously all utilized CNNs as their primary network for feature extraction, the ViT (Vision Transformer) has also been explored. In a study employing a hybrid model that combines CNN and ViT as the backbone, the prediction accuracy for classifying BMS cells surpassed that of other CNN-based models.^76^

Beyond classifying cell types in BMS, these techniques can also differentiate cells of the same type with varying morphologies. This is particularly relevant for identifying mutations in acute myeloid leukemia (AML) blasts, which can present distinctive morphological features. For instance, blasts with NPM1/FLT3-ITD mutations often exhibit unique cup-shaped nuclei.^77^ Thus, DL models hold potential for predicting specific mutations by analyzing the morphology of blasts alone. Eckardt *et al.* implemented Faster R-CNN for the segmentation of nucleated cells, followed by using a ResNet model to predict NPM1-mutated blasts, achieving an accuracy of 0.86.^78^ Meanwhile, Kockwelp *et al.* sought to classify five distinct AML types: CBFB::MYH11, NPM1 mutation, FLT3-ITD mutation, AML with myelodysplastic changes, and a fifth category, favorable risk AML. The first four categories are associated with specific morphological features – such as atypical eosinophils, cup-shaped nuclei (with and without NPM1 mutation), and dysplastic changes, respectively –while the fifth lacks uniform morphological characteristics.^79^ Despite the emphasis on high-quality segmentation of blasts, their classification model was relatively straightforward, employing an 18-layer ResNet. This approach led to high accuracy for CBFB::MYH11 (AUC 0.9) and NPM1 mutations (AUC 0.88), but the performance for the other three categories was lower, with AUCs ranging from 0.6 to 0.7.^79^ Although these results are promising, their applicability is limited to mutations with distinctive morphologies, and further validation using external datasets is necessary.

A special use case of automated cytomorphology recognition is in imaging flow cytometry (IFC), which enhances traditional flow cytometry by integrating cameras. This allows for the capture of brightfield, darkfield, and fluorescent images of individual cells.^80^ Since the cell images are already individually segmented as cells move past the camera one by one, DL-based models can be directly applied to these images.^81^ In a study aiming to identify WBC subtypes using stain-free IFC images, both traditional ML and CNN-based models achieved comparably accurate results.^82^

### DL in cytometry

The analysis of multiparameter flow cytometry (MFC) or mass cytometry (CyTOF) requires substantial expertise and the results are not always reproducible.^83^ Moreover, MFC raw data, essentially a vast table with rows and columns representing different cells and the fluorescent intensity of various markers respectively, is high-dimensional and well-suited for ML methods. To identify individual cell labels, either a linear layer or an MLP can be utilized to integrate the information from each marker, mirroring the process of determining cell types through the combination of CD markers. Once trained, the neural network can be applied to the whole sample to classify each cell. Therefore, this method can be used to determine minimal residual disease (MRD). In a study aiming to detect chronic lymphocytic leukemia (CLL) MRD, a three-layer MLP was trained, which had over 99% sensitivity and specificity for identifying CLL cells from normal lymphocytes.^84^ A key limitation of this approach is the need for manual annotation of individual cell labels to train the model, which is extremely labor-intensive.

Another common method for training DL models in cytometry involves weakly supervised learning, where only the sample-wide label is available (e.g., leukemia vs. no leukemia). This sets up a multiple instance learning (MIL) situation, where the information of individual cells needs to be combined to determine the label of the whole sample. In the *CellCnn* model, each cell’s markers undergo a linear transformation (convolution) with multiple kernels, after which a max pooling layer aggregates cells’ information by selecting the maximum value across all cells to predict the sample’s label.^85^ (Figure 5B) This method enables the model to differentiate between samples from healthy BM and those from an AML patient with an MRD of 0.01%.^85^ A related model, *DeepCellCNN*, employs two convolutional layers instead of one, as in CellCnn, resulting in marginally better outcomes.^86^ Performance can be further enhanced by adopting a new prediction objective: instead of predicting a binary label (e.g., leukemia vs. no leukemia), the model predicts the percentage of events, such as the proportion of leukemia cells within a sample.^87^ Another variation of the traditional CellCnn model incorporates an attention module to aggregate cell information instead of max pooling.^88^ This adaptation has achieved over 90% accuracy in diagnosing acute leukemia and distinguishing between various types of acute leukemia. However, a notable limitation of this approach is that calculating attention scores across hundreds of thousands of cells is computationally demanding and resource-intensive.

## Deep Learning at the Tissue Level

### Challenges in Whole Slide Image Application

Compared to traditional computer vision tasks, applying DL models to interpret whole slide images (WSIs) presents unique challenges. First, WSIs are exceptionally large, typically measuring around 100,000 x 100,000 pixels,^89^ in stark contrast to the much smaller input size of 224 x 224 pixels used in CV datasets like ImageNet.^90^ To achieve computational efficiency, DL models often necessitate dividing WSIs into smaller patches, also known as tiles, containing only hundreds to thousands of pixels in each dimension. This allows for pixel-level calculations to be conducted on each individual patch. Consequently, most studies on WSIs adopt a two-stage methodology: initially, a feature extractor, typically a CNN, analyzes the pixels within individual patches to generate patch embeddings. Subsequently, these embeddings are integrated using aggregation algorithms for WSI-level predictions. (Figure 5C) A CNN model pre-trained on a general image dataset, like a ResNet with ImageNet, can be effectively transferred for feature extraction from patch pixels of pathology images.^25^ Interestingly, CNNs trained specifically in histopathology datasets show only marginal enhancement in feature extraction compared to those trained on general image datasets like ImageNet.^91,92^ Nevertheless, these histopathology-specific feature extractors may be either trained in a fully supervised version,^92^ where the labels for each patch are required, or more commonly through unsupervised training using contrastive learning.^91,93^ Furthermore, self-attention-based feature extractors, such as the Vision Transformer (ViT) and Swin Transformer, have been recently applied in WSI analysis for patch feature extraction.^94,95^

The second challenge is in applying DL to WSIs is the scarcity of curated training samples. The expertise required for WSI annotation limits the number of qualified annotators, making the process challenging. Initially, training models necessitated annotations for every single patch, a process that was exceedingly labor-intensive.^96,97^ This issue has been partially addressed through weakly-supervised training methods, which rely on slide-level rather than patch-level annotations, greatly reducing the annotation burden. In recent years, there have been significant efforts to create publicly accessible histopathology datasets, facilitated by challenges like PANDA^98^ and CAMELYON,^99^ or through open datasets such as TCGA.^100^ Additionally, there have been innovative attempts to curate data on social media platforms, like X, where clinicians have shared over 200,000 de-identified histopathologic images, contributing to the growing availability of data for research and model training.^101^ In hematology, there is a notable scarcity of large datasets of bone marrow WSIs, possibly because WSIs serve only auxiliary roles in the diagnosis of most hematologic malignancies. In practice, typically only hundreds of bone marrow WSIs are utilized for training DL models, highlighting the challenge of limited data availability in this specific area of medical research.^102^

Third, WSI is inherently patchy – only certain sections of a slide might show pathological changes, while the rest could appear normal. This scenario fits into multiple instance learning (MIL), where the diagnosis for the whole slide is based on a subset of these patches. Therefore, various techniques have been employed to aggregate features from individual patches. A commonly used method is mean pooling, which involves calculating the average features of all patches to make a single prediction. However, this approach struggles with imbalanced instances, where the majority may be normal and only a few patches show pathological changes, because it dilutes the significance of abnormal patches, overshadowing key pathological information with predominant normal findings. A solution is top-K pooling, selecting the top K patches with the highest feature scores to label the slide.^96,103^ However, this approach trains the model using only a few patches per slide (K number of patches), necessitating more WSIs to match the performance of fully supervised models.^103^ A more refined approach involves assigning varying weights, referred to as attention scores, to different patches. These scores are analogous to their diagnostic importance and can be learned through training.^24^ (Figure 5D) This method, known as attention-based MIL, effectively integrates the features from all patches. Another benefit of this method is interpretability: by indicating the importance of each patch in contributing to the diagnosis through weights, mapping a heatmap of these weights onto the spatial locations of the original patches visually demonstrates the significance of each region to the overall slide-level diagnosis. A model using this attention-based MIL, *CLAM*, achieved an AUC exceeding 0.95 in classifying subtypes of various solid tumors, even when trained on fewer than a thousand samples.^25^ However, a limitation of the attention-based MIL approach is its lack of context awareness: each patch processes information independently without access to the contextual data of adjacent patches. This limitation is critical in scenarios like *hypoplastic* myelodysplastic syndrome (MDS). In such cases, patches containing dysplastic cells may indicate MDS, but accurately diagnosing requires combining this feature with the context of the surrounding cellularity. Information from other patches can be incorporated through RNN^103,104^ or self-attention-based models^95,105,106^to address the issue of context awareness in attention-based MIL. Self-attention can be directly applied to all patches like ViT, but this method is highly computationally demanding due to the vast number of patches involved.^106^ One strategy to mitigate this is by increasing the pixel count per patch, thereby reducing the total number of patches.^105^ However, this adjustment might compromise the level of detail in feature extraction from the patches. An alternative and more efficient method employs a hierarchical structure that aggregates patches from small regions to medium-sized windows and finally to the entire slide level.^95^ (Figure 5E) This context-aware model demonstrates enhanced performance compared to traditional MIL models, though it still comes with a markedly increased computational cost.

### DL in histopathology

Lymph node (LN) biopsy and bone marrow (BM) biopsy are the two most common histopathological samples in hematology (Table 1). These samples exhibit unique characteristics compared to biopsies from solid tumors. First, the presence of lymphoma in a LN or leukemia in a BM tends to be more homogenous, making a patch-level representation often sufficient for classifying the WSI. Second, cellular morphology in LN and BM samples plays a more significant role in disease diagnosis than it does in solid tumors, requiring models to place greater emphasis on morphological features. Furthermore, the cell distribution in BM biopsies can be particularly indicative of certain diseases, such as aplastic anemia and myeloproliferative diseases, with changes in cellularity and disruption of the normal architecture being key diagnostic criteria. Therefore, DL models in hematology have been tailored to focus on these characteristics.

**Table 1.** Studies using deep learning in hematology whole slide imaging interpretation

**Biopsy sample**	**Clinical Task**	**Training size**	**DL model: Patch Feature Extractor**	**DL model: Patch Feature Aggregator**	**Testing dataset**	**Testing results**	**References**
LN	Differentiate DLBCL, BL, SLL, and benign	128	CNN on manually cropped area	None	Internal	Accuracy 95%	Achi et al., 2019^107^
LN	Differentiate DLBCL from various benign and malignant LN samples	1,754	Majority-voting of 17 CNNs on manually cropped area	None	External	Accuracy >99%	Li et al., 2020^108^
LN	Differentiate DLBCL, FL, and benign	388	CNN on manually cropped area	None	Internal	Accuracy 90% AUC 0.95	Miyoshi et al., 2020^109^
LN	Differentiate DLBCL, SLL, and benign	629	CNN on manually cropped area	None	External	Accuracy 96%	Steinbuss et al., 2021^110^
LN and other biopsy sites	Predict *MYC* rearrangement on H&E stained DLBCL WSIs	287	CNN	Not clearly specified	External	Accuracy 74% AUC 0.83	Swiderska-Chadaj et al., 2021^111^
LN	Differentiate FL and benign hyperplasia	378	CNN	Mean pooling	External	AUC 0.66	Syrykh et al., 2020^112^
Skin	Annotate CD30+ regions on CD30-stained WSIs to diagnose CD30+ LPD	28	CNN	Local self-attention, sum pooling	Internal	Accuracy 96% AUC 0.99	Zheng et al., 2023^113^
BM	Predict mutations on H&E stained MDS WSIs	236	Pretrained CNN	Mean pooling	Internal	AUC varies on mutations, as high as 0.94	Bruck et al., 2021^114^
BM	Differentiate AML, CML, ALL, CLL, and MM	129	Pretrained CNN	Attention	External	Accuracy 94% AUC 0.97	Wang et al., 2022^115^
BM	Differentiate ET and prePMF	226	Pretrained CNN	Attention	Internal	Accuracy 92% AUC 0.90	Srisuwananukorn et al., 2023^116^
BM	Differentiate AL, MM, LPD, and normal	556	Pretrained YOLO for cell detection and feature extraction	Attention	Internal	Average F1 score 0.57	Mu et al, 2023^117^

DL, deep learning. LN, lymph nodes. DLBCL, diffuse large B-cell lymphoma. BL, Burkitt’s lymphoma. SLL, small lymphocytic lymphoma. CNN, convolutional neural network. FL, follicular lymphoma. AUC, area under curve. WSI, whole slide image. LPD, lymphoproliferative disease. BM, bone marrow. MDS, myelodysplastic syndrome. AML, acute myeloid leukemia. CML, chronic myeloid leukemia. ALL, acute lymphoblastic leukemia. CLL, chronic lymphocytic leukemia. MM, multiple myeloma. ET, essential thrombocythemia. prePMF, prefibrotic primary myelofibrosis. AL, acute leukemia.

In the realm of DL tasks in LN-derived WSIs, the primary focus of most studies is to differentiate among various types of lymphomas and related conditions. This includes distinguishing aggressive lymphomas such as diffuse large B-cell lymphoma (DLBCL) and Burkitt’s lymphoma (BL), from indolent lymphomas like follicular lymphoma (FL) and small lymphocytic lymphoma (SLL), as well as from reactive hyperplasia or normal lymph nodes, using hematoxylin and eosin (H&E) stained slides.^107–110^ In a departure from this common objective, one study sought to predict *MYC* rearrangement in DLBCL WSIs using H&E staining but achieved low accuracy.^111^ The unique cytomorphology of different lymphomas means that features extracted from just a single patch can often accurately diagnose the WSI. Indeed, most studies have applied a CNN to a manually selected patch, achieving diagnosis accuracies over 90%. In one study, 17 CNN models were fined-tuned to differentiate between DLBCL and non-DLBCL samples using cropped images of approximately 1,000x1,000 pixels.^108^ To improve the results, a “majority voting” trick was used, wherein each model’s individual prediction contributed to a final diagnosis based on the majority consensus among the models. Only one published study employs the conventional feature extractor-aggregator framework for analyzing WSIs. That research aimed to differentiate between FL and benign follicular hyperplasia (FH) using H&E-stained WSIs. The study began by training a CNN to distinguish FL and FH at the patch level, then implemented mean pooling to assign a label to the entire WSI.^112^ However, the model’s performance on an external testing dataset resulted in an AUC of just 0.66, indicating limited generalization ability.

Another study focused on differentiating lymphomatoid papulosis from primary cutaneous anaplastic large-cell lymphoma using CD30-stained skin WSIs based on the extent of CD30-positive cell involvement.^113^ To effectively incorporate information from adjacent patches, the authors implemented a local self-attention mechanism. This technique allowed for integrating the feature vector from the central patch with those from surrounding patches. Consequently, the overall percentage of CD30-positive regions within the WSI was determined by aggregating all the positively identified patches.

Several studies have also explored the use of DL in interpreting BM WSIs, covering a variety of tasks from distinguishing between different disease types to predicting mutations through morphological features.^114–117^ Commonly, these studies employ a CNN as a feature extractor, followed by an aggregator to compile patch-level features into slide labels. In a work focused on predicting mutations associated with MDS, patch features were extracted directly using CNN models pre-trained on the ImageNet dataset without any fine-tuning for histopathological data.^114^ The feature vectors from each patch were then condensed into a single value using an MLP tailored for various mutations. The overall label for the WSI — indicating the presence or absence of specific mutations — was determined by averaging these values across all patches. Despite the simplicity of this model architecture, it achieved high AUC scores, exceeding 0.90 for certain mutations, such as *ASXL1* and *TET2*. Additionally, attention-based MIL methods have also been applied. In a study to distinguish between hematologic malignancies using bone marrow smear WSIs, patch features were extracted using a CNN model pre-trained on ImageNet. This was followed by the application of the CLAM framework to assign slide-level labels.^115^ This approach demonstrated a 94% accuracy rate in identifying various hematologic malignancies using an external test dataset. Another study aimed to distinguish essential thrombocythemia from prefibrotic primary myelofibrosis. It first used a CNN pre-trained on histopathological images to extract features, then applied the CLAM framework to integrate the features of individual patches.^116^ This model achieved 92% accuracy in differentiating the two conditions. One study employed an attention-based aggregator different from CLAM to differentiate acute leukemia, multiple myeloma, and lymphoproliferative disease from bone marrow WSIs.^117^ Utilizing the YOLO object detection model, individual cells were segmented and their features extracted. Next, an attention-based aggregating algorithm, known as Hopfield pooling,^118^ was applied to integrate these features by assigning weights to individual cell images. However, the performance was modest: with internal testing datasets, the F1 score, an accuracy indicator, was only 0.57.

Overall, the field of digital pathology has witnessed significant advancements, paving the way for innovative applications in hematology. Despite these achievements, the integration of DL techniques in hematology primarily relies on established methodologies. While studies demonstrate the potential of DL in analyzing various hematological conditions, the adoption of newer, more sophisticated DL models is still in its nascent stages. Moreover, challenges in model generalization and modest performance in external datasets highlight the need for ongoing research and development.

## Deep Learning at the Patient Level

### DL in curated clinical data

DL models, particularly MLPs, can be utilized to predict clinical outcomes in hematology using curated patient data. For example, one study employed an MLP to combine patient demographics with laboratory test results to predict the likelihood of successful donor hematopoietic stem cell mobilization.^119^ Another study trained an MLP to predict the survival status at the last follow-up of patients with DLBCL based on 740 gene expression profiles.^120^ Despite the capabilities of DL models, comparisons with traditional regression and classic ML methods reveal minimal improvements in prediction accuracy, and, in some instances, they perform worse than classic ML methods. This discrepancy arises probably because, although DL models can process a wide range of variables, only a few significantly impact clinical outcomes. Consequently, DL models’ advantage in handling complex data types remains underutilized. For example, in a study predicting 100-day non-relapse mortality for over 25,000 patients undergoing allogeneic hematopoietic stem cell transplantation, logistic regression, tree-based classic ML methods, and an MLP were used with 23 selected variables.^121^ The study demonstrated that all methods achieved similar AUC scores, highlighting that incorporating just 3 to 5 key variables was sufficient to reach near-maximal AUCs, underscoring the limited benefit of DL models in this context.

### DL in electronic health records

**Figure 6.**
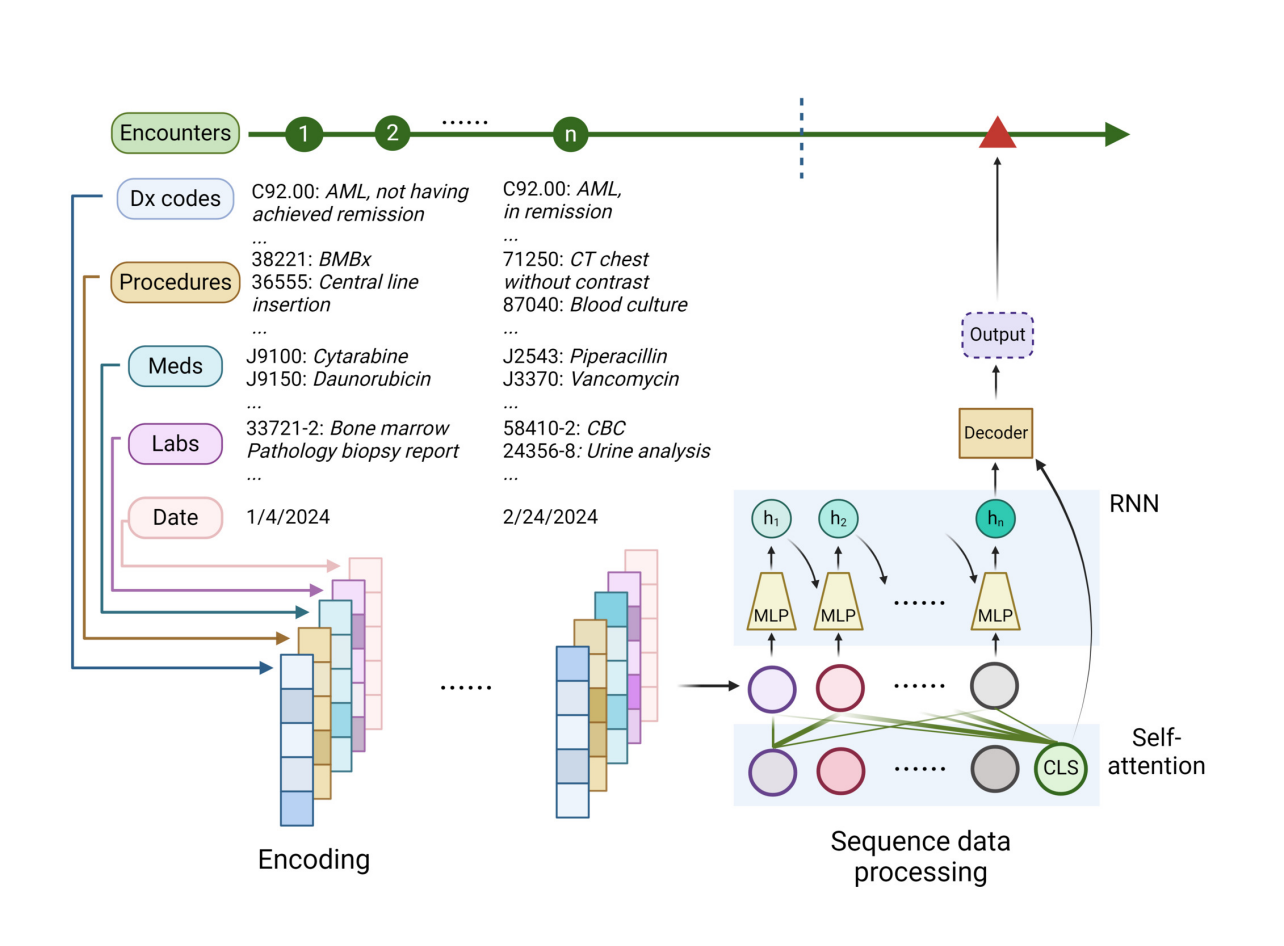
A framework for deep learning in electronic health records (EHRs). Deep learning techniques are used to process and predict administrative medical codes in EHRs. The codes are encoded into numerical representations, and deep learning models like recurrent neural networks (RNNs) or self-attention models are employed to extract meaningful patterns from the sequential data. These models generate a latent representation (denoted as “h_n_” for RNNs or “CLS” for self-attention models in the figure) that encapsulates the salient features of the entire EHR sequence. This latent representation is then used to predict the administrative codes for future clinical encounters.

An alternative approach to predicting clinical events involves applying DL models to non-curated, patient-level electronic health records (EHR) data. In this method, each clinical encounter is treated as a data point comprising structured medical codes such as the International Classification of Disease (ICD) diagnosis codes, medication codes, procedure codes, and laboratory codes. This collection of clinical encounters forms sequence-type data, encapsulating a patient’s medical history. Unlike the analysis of curated data, this approach additionally leverages temporal and longitudinal information in the EHR, providing a comprehensive view of patient health over time. The analysis process typically involves three steps. Initially, the medical codes associated with each clinical encounter and their timestamps are encoded into numerical representations. Subsequently, DL models process these sequence-type data, mapping them into a latent space. This step is analogous to summarizing a patient’s medical history or clinical trajectory. Finally, a prediction mechanism, usually an MLP, operates on this latent space to produce the prediction outcome. (Figure 6) In the embedding process, although many models adopt the random “one-hot” encoding, the use of learned embeddings — where similar medical concepts have closely related embeddings — can enhance model performance.^122^ As for transforming embeddings into latent representations, RNNs are commonly used due to their proficiency in processing sequence-type data.^123^ For instance, the *DoctorAI* model inputs medical codes from past encounters into an RNN, creating a contextualized representation of the patient’s medical history, which is then utilized to predict medical diagnoses and medication codes for the subsequent visit.^124^ Another study expanded this approach by including clinical notes, tokenizing each word in the free texts, and combining them with medical codes.^125^ This enriched input set was used to generate predictions for in-hospital mortality, readmission rates, and the length of hospital stays, demonstrating the potential of integrating diverse data types for more accurate health outcome predictions. The advent of the Transformer architecture has led to a shift towards self-attention-based DL models in predictive modeling for EHR.^126^ A notable example is the *BEHRT* model, which treats diagnosis codes from each visit as words in a sentence.^127^ Its pre-training objective involves predicting masked diagnosis codes, mirroring BERT’s training methodology. An MLP prediction head is trained on the model’s outputs for tasks such as predicting diagnosis codes for future visits.

While the above methods remain in the proof-of-concept phase, their deployment in hematology remains limited. Notably, a study focusing on predicting the two-year survival of patients with AML, based on the first six months of laboratory and bone marrow histological data, employed a heterogeneous graph transformer model.^128^ This approach achieved an AUC of 0.76 on an external testing dataset, demonstrating performance comparable to the predictions based on the European LeukemiaNet (ELN) 2022 criteria, even without incorporating molecular and cytogenetic information.^129^

However, these studies have several limitations. First, while these models often show strong performance within their training datasets, achieving AUC values often exceeding 0.90, testing on external independent datasets is seldom conducted. A systematic review found that only 3 out of 81 studies (3.7%) conducted external testing.^130^ This scarcity of external validation raises questions about the models’ generalizability. Furthermore, models trained on private datasets often do not disclose their parameters for privacy reasons, complicating these methods’ external evaluation. Third, the structured medical codes used in training these models are frequently criticized for inaccuracies and lack of granularity.^131^ Thus, validation against original medical records is crucial to verify these results. Fourth and most importantly, some prediction tasks are clinically implausible due to the complex and multifaceted nature of clinical events, which are influenced by numerous unmeasurable variables not captured by medical codes. For instance, it is unrealistic to predict the specific reason for a future hospital admission with high confidence based solely on past medical encounters. Additionally, certain clinical events, such as the onset of pancreatic cancer, are sporadic and minimally influenced by a patient’s medical history. Indeed, despite known associations between several non-specific environmental risk factors (e.g., smoking and obesity) and an increased risk of pancreatic cancer,^132^ no definitive clinical factor has been identified as a direct cause of the cancer. A study attempting to predict pancreatic cancer occurrence up to 36 months in advance using DL models trained on medical codes illustrates this point. The models exhibited very low precision and recall, barely reaching 1%.^133^ Although the specificity was reported to be near 100%, this likely resulted from imbalanced labels in the training datasets, where the vast majority of patients did not have pancreatic cancer. This could have led to the models to “cheat” by simply predicting that no patients had pancreatic cancer. In such a scenario, the models would correctly identify most patients without pancreatic cancer, resulting in high specificity, but would fail to identify the few patients who actually had the disease, leading to low precision and recall.

### DL in clinical notes

Prior to the prevalence of Transformer-based LLMs, RNNs and CNNs were commonly employed for semantic analysis in clinical notes. In a study aimed at identifying bleeding events from EHR clinical notes, both a CNN and an RNN were utilized to assess individual sentences for descriptions of bleeding, achieving an accuracy rate of 90%.^134^ The introduction of LLMs has enabled the execution of more complex tasks. Notably, state-of-the-art models like GPT-4 and Med-PaLM 2 have demonstrated the capability to accurately answer US Medical Licensing Exam (USMLE) style questions with an accuracy rate exceeding 85%.^135,136^ This performance underscores their capability to comprehend and analyze complex medical scenarios. Various LLM-assisted clinical tasks have been proposed.^137^ LLMs are notably effective in generating clinical notes from doctor-patient conversations, with a study highlighting that notes generated by GPT-4 were as preferred as those written by humans.^138^ Furthermore, LLMs have shown proficiency in identifying patient eligibility for clinical trials based on clinical notes, with a study utilizing GPT-3.5 revealing high accuracy rates of 86% and 84% for matching inclusion and exclusion criteria, respectively.^139^ Additionally, LLMs have been leveraged to enhance the readability of clinical documentation. A study showed that the systematic implementation of ChatGPT in a hospital significantly improved the readability of informed consent documents, such as those for bone marrow biopsies, making them more accessible to the average American.^140^ Lastly, LLMs can also help decision-making based on clinical notes. A study compared the responses of hematologists and various LLMs regarding hematopoietic stem cell transplantation eligibility, donor selection, and conditioning regimens across six clinical cases of patients with hematological malignancies.^141^ The study showed that LLMs exhibited strong performance in determining patients’ eligibility and selecting donors, yet they fell short in recommending appropriate conditioning regimens.

In addition to tasks related to clinical notes, LLMs hold potential in various aspects of patient care, such as creating medical chatbots for triage, question answering, medication management, automating medical history taking, and generating medical reports from scans or histopathological images.^142,143^ These applications will likely be developed initially in a general medical setting before being adapted and implemented in specialized fields like hematology. While promising, future research should address the challenges of robustness, explainability, and the ethical implications associated with using LLMs in healthcare.^144,145^

## Conclusion

DL has demonstrated diverse applications across various domains of hematology. At the molecular level, DL models have significantly advanced multi-omics data analysis and protein structure predictions. For cells and tissues, DL techniques enable the automation of cytomorphology analysis, interpretation of flow cytometry data, and diagnosis from whole slide images. Additionally, DL shows promise in predicting clinical outcomes using patient data and electronic health records. The advent of LLMs further facilitates complex tasks such as generating clinical notes and supporting decision-making processes.

Despite these advancements, DL faces specific challenges, including the need for larger, curated datasets, enhanced model interpretability, and improved generalizability. These challenges are particularly pronounced in hematology, where the adoption of new DL models is notably slower than in other medical fields. Future endeavors should develop hematology-tailored models, integrate multimodal data, and ensure generalizability. Interdisciplinary collaboration between hematologists, computer scientists, and regulatory bodies is vital to unlocking DL’s full potential in transforming hematological research and clinical care.

We stand on the brink of a transformative period, marking the advent of a more profound integration of DL into the standard practices of hematology. This integration could significantly enhance patient care by providing more accurate diagnoses, personalized treatment plans, and improved patient outcomes. However, to fully realize these benefits, it is imperative that the upcoming generation of hematologists not only become adept at employing these advanced technologies but also gain a comprehensive understanding of the underlying principles of DL.

### Conflicts of Interest

None

### Author Contributions

Concept and design: All authors

Manuscript writing: All authors

Final approval of manuscript: All authors
